# Intracranial calcifications in *DEGS1*-Related Leukodystrophy: a potentially under-recognised neuroimaging feature

**DOI:** 10.1007/s10072-026-09147-5

**Published:** 2026-06-09

**Authors:** Ylenia Vaia, Neena Kim, Sharmila Jeyasingh, Sniya Sudhakar, Kshitij Mankad, Asthik Biswas, Ata Siddiqui, Helen Mundy, Tammy Hedderly, Rahul R. Singh

**Affiliations:** 1 Department of Biomedical and Clinical Sciences, Neuroscience Research Centre, University of Milan, Milan, Italy; 2 Children’s Neurosciences, Evelina London Children’s Hospital at Guys and St Thomas’ NHS Foundation Trust, Wstminster Bridge Road, London, SE1 7EH UK; 3 Department of Neurology, Great Ormond Street Hospital for Children, London, UK; 4 Department of Paediatrics, Royal Alexandra Children’s Hospital, Brighton, UK; 5 Department of Radiology, Great Ormond Street Hospital for Children, NHS Foundation Trust, London, UK; 6 Department of Neuroradiology, Evelina London Children’s Hospital at Guys and St Thomas’ NHS Foundation Trust, London, UK; 7 Department of Inherited Metabolic Disease, Evelina London Children’s Hospital, London, UK; 8 KCL Faculty of Life Sciences and Medicine, London, UK

**Keywords:** hypomyelinating leukodystrophy, Intracranial calcification, Sphingolipid biosynthesis

## Abstract

Pathogenic variants in *DEGS1*, encoding a sphingolipid desaturase critical for ceramide biosynthesis, disrupt sphingolipid homeostasis and oligodendrocyte function, leading to abnormal myelination. We report an infant with genetically confirmed *DEGS1*-related leukodystrophy (homozygous c.337A > C, p.Asn113His) who presented with abnormal eye movements and early-onset developmental arrest. This was accompanied by axial hypotonia, severe feeding difficulties, and refractory epilepsy, including epileptic spasms with modified hypsarrhythmia. Brain MRI demonstrated diffuse abnormal myelination per age, thin corpus callosum, and cerebellar involvement. Notably, susceptibility-weighted imaging suggested cerebellar white matter calcifications, which were confirmed on brain CT, alongside punctate supratentorial calcifications, an atypical finding for *DEGS1*-related disease. This case expands the neuroimaging phenotype of *DEGS1*-related leukodystrophy and highlights intracranial calcifications, particularly within the cerebellum, as a potential diagnostic clue in the differential diagnosis of hypomyelinating disorders with conatal onset. Our findings also underscore the severe clinical course associated with DEGS1 deficiency, including profound developmental impairment, early-onset epilepsy, and persistent feeding difficulties.

## Introduction

Hypomyelinating leukodystrophies comprise a genetically heterogeneous group of disorders characterised by impaired formation or maintenance of myelin. The *DEGS1* gene encodes delta 4-desaturase, sphingolipid 1, that catalyses the conversion of dihydroceramide to ceramide, a critical step in sphingolipid biosynthesis [1]. Impairment of this enzyme leads to dihydroceramide accumulation, disruption of sphingolipid homeostasis, and disturbed intracellular signaling, ultimately interfering with oligodendrocyte function and myelin formation [1, 2]. Pathogenic variants in *DEGS1* were first identified in 2019 as a cause of hypomyelinating leukodystrophy (HLD18), resulting from disruption of ceramide synthesis and sphingolipid homeostasis ^1^ Affected individuals typically present in early infancy with developmental arrest, abnormal eye movements, progressive spasticity, epilepsy, and severe failure to thrive [1, 3] Neuroimaging usually demonstrates diffuse hypomyelination, involving supratentorial and infratentorial structures. [1, 3]

Here, we report an infant with genetically confirmed *DEGS1*-related leukodystrophy harbouring a homozygous c.337A > C variant, presenting with white matter abnormalities and intracerebral calcifications, most prominently involving the cerebellar white matter. This finding highlights a potentially important diagnostic clue that may aid early recognition of this rare disorder.

## Case report

This male child was referred at 6 weeks of age for evaluation of abnormal eye movements, delayed development and feeding difficulties. He was born at term following an uncomplicated pregnancy and delivery. Developmental milestones were profoundly delayed. By 8 weeks, he had not achieved a social smile or vocalisation, indicating early global developmental arrest. Neurological examination at that time demonstrated multidirectional nystagmus, absent visual fixation and axial hypotonia with poor head control. In addition, bilateral sensorineural hearing loss was confirmed. He had failure to thrive, leading to nasogastric tube placement by 10 weeks of age. By 4 months of age, neurological examination demonstrated minimal spontaneous movement, with emerging dystonia, limbs spasticity and clonus. Prominent nystagmus persisted. At 7 months of age, he developed asymmetric epileptic spasms together with tonic seizures. Electroencephalography demonstrated modified hypsarrhythmia. Treatment with vigabatrin was initiated and resulted in partial benefit on the epileptic spasms. Feeding difficulties remained severe, and a gastrostomy tube was placed at 3 years. Over time, there has been minimal neurodevelopmental progress, and the clinical course has been complicated by frequent chest infections associated with oxygen desaturation, requiring several hospital admissions. At the most recent evaluation at 6 years of age, the child continues to exhibit profound developmental impairment. He has not achieved trunk control and only communicates through facial expressions; he still presents refractory epilepsy and severe dystonia. He is currently receiving palliative care support.

### Neuroimaging

**Fig. 1:**
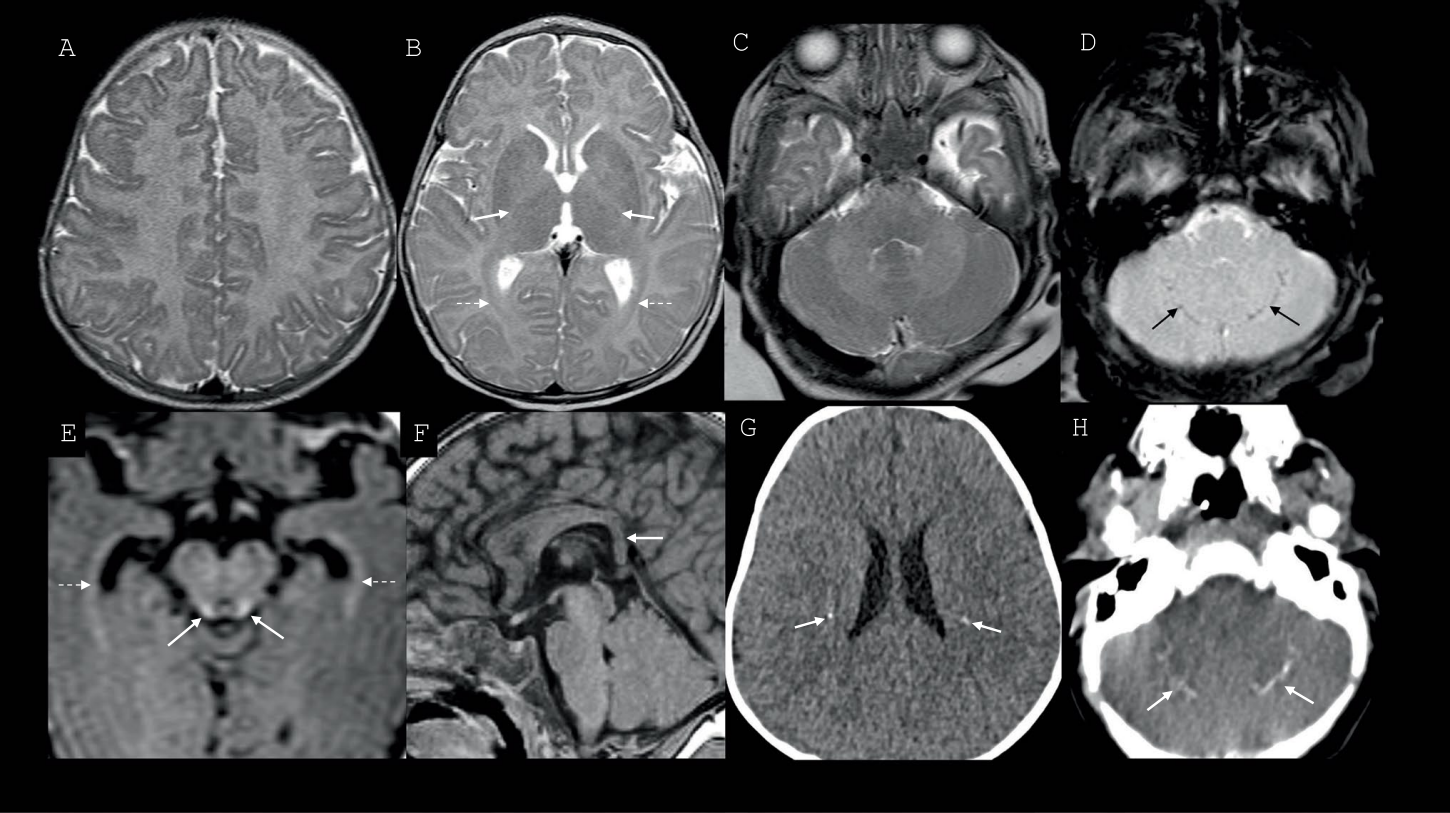
Brain MRI and CT findings at 4.5 months of life A-C: Axial T2-weighted images at the level of the centrum semiovale (A), deep gray nuclei (B) and posterior fossa (C) show diffuse abnormal hyperintensity consistent with deficient myelin deposition for age. Note relative sparing of the posterior limbs of the internal capsule (arrows, B), and optic radiations (dashed arrows, B) which demonstrate T2 hypointensity (also see E and F). D: Axial T2* weighted sequence shows symmetric curvilinear hypointensities involving the cerebellar white matter (arrows) consistent with calcifications (see G and H). E: Axial T1-weighted image shows myelination within the optic radiations (dashed arrows) and inferior colliculi (arrows). F: Sagittal T1-weighted sequence shows a thin posterior corpus callosum (arrow). G and H: Axial CT scans confirm punctate calcifications involving the posterior corona radiata (arrows, G), and curvilinear calcifications involving the cerebellar white matter (arrows, H)

Brain MRI performed at 4.5 months of age demonstrated markedly deficient myelin deposition, involving both supratentorial and infratentorial white matter and brainstem, with relative sparing of the posterior limbs of the internal capsules, optic radiations and inferior colliculi. (Fig. 1A,B,C,E) The corpus callosum was mildly thinned posteriorly. (Fig. 1F) Symmetric curvilinear hypointensities were observed on T2* gradient echo imaging (GRE), which were confirmed to correspond to curvilinear and punctate calcifications on CT imaging. Punctate calcifications were also noted involving the corona radiata bilaterally. (Fig. 1D, G, H).

### Genetic findings

Trio whole-exome sequencing (WES) identified a homozygous missense variant in *DEGS1* (NM_003676.3: c.337A > C, p.Asn113His), of biparental inheritance. The variant affects a highly conserved residue within the sphingolipid desaturase domain of the DEGS1 protein, a region critical for normal enzyme function in sphingolipid metabolism. Based on available evidence, including consistency with the clinical and neuroimaging phenotype, the variant was classified as pathogenic.

## Discussion

We describe a child diagnosed with the severe early-onset phenotype of *DEGS1*-related leukodystrophy, characterised by profound developmental impairment, early abnormal eye movements, epilepsy, and severe feeding difficulties.

To date, 37 patients with *DEGS1*-related leukodystrophy have been reported [4]. The clinical presentation of our patient closely resembles previously reported cases, with early developmental arrest, prominent nystagmus, severe feeding difficulties, and early-onset epilepsy. The identified p.(Asn113His) variant, affecting a highly conserved residue within the catalytic domain of the DEGS1 protein, is consistent with the severe phenotype observed in our patient, as severe early-onset disease has been described in association with both missense and loss-of-function variants that disrupt the enzyme’s catalytic activity. [1, 3]

Neuroimaging features reported in *DEGS1*-related leukodystrophy include hypomyelination, periventricular white matter T2/FLAIR hyperintensities, thinning of the corpus callosum, and cerebellar atrophy [1, 3, 4] In keeping with this spectrum, our patient demonstrated inappropriate myelination pattern per age, with the additional finding of calcifications involving the cerebellum and corona radiata. Intracranial calcifications have a broad differential diagnosis, encompassing both acquired and genetic causes [5, 6]. The coexistence of abnormal myelination and parenchymal calcifications has been described in conditions such as Cockayne syndrome, AGS-genes related interferonopathies, and more recently in a case of GM1 gangliosidosis [5, 7, 8]. The morphology of calcifications in these disorders is variable, ranging from coarse and punctate to linear, and may involve the basal ganglia, thalami, cortico-subcortical junction, brainstem, and cerebellum [9]. However, these patterns are often non-specific, and meaningful diagnostic discrimination relies on the integration of calcification distribution and morphology with broader imaging findings and systemic features, such as the presence of a positive interferon signature and basal ganglia involvement in AGS [10], a progeroid phenotype and progressive microcephaly in Cockayne syndrome [11], and coarse facies with a cherry red spot in GM1 gangliosidosis. [12]

To our knowledge, the distinct pattern of symmetric curvilinear calcification along the subcortical cerebellar white matter observed in this case has not been previously reported. Pant et al. described intracranial calcifications in 4 out of 19 patients with *DEGS1*-related leukodystrophy; however, the morphology and distribution of these calcifications were not characterised, and no representative images were provided. The biological mechanism underlying calcifications in this context remains uncertain. Disruption of sphingolipid metabolism, as occurs in DEGS1 deficiency, may contribute to cellular injury and secondary mineral deposition within affected white matter. [11] Interestingly, this may represent a broader vulnerability of white matter to sphingolipid dysregulation, as seen in lysosomal sphingolipidoses such as Krabbe disease, which can also feature white matter calcifications, despite the metabolic defect involving lysosomal rather than endoplasmic reticulum sphingolipid pathways [13, 14]. However, in Krabbe disease, the presence of peripheral neuropathy and elevated CSF protein levels may support the differential diagnosis. [15]

In addition, in our case, normal renal profile, serum calcium, phosphate, vitamin D and parathormone levels allowed us to exclude secondary metabolic causes of intracranial calcifications, and other common aetiologies, including congenital infections and acquired vascular or inflammatory processes, were also not supported by the clinical and laboratory findings. Therefore, the observed calcifications were more likely to be directly associated with the underlying *DEGS1*-related pathology. Importantly, recognition of this imaging feature may help in the radiological differential diagnosis of hypomyelinating leukodystrophies, particularly in cases with connatal or early infantile presentations.

## Conclusion

*DEGS1*-related leukodystrophy should be considered in infants presenting with early developmental arrest, nystagmus, epilepsy, and inappropriate myelination per age. Intracranial calcifications, particularly involving the cerebellar white matter, may serve as an additional imaging clue, further expanding the phenotypic spectrum of *DEGS1*-related leukodystrophy.

## Data Availability

Written informed consent for publication of this case report and any accompanying images was obtained from the patient’s parent. All relevant data supporting the findings of this case report are contained within the manuscript.
